# Is the size of the transforaminal lumbar cage a risk factor for cage subsidence? a retrospective cohort study

**DOI:** 10.1007/s10143-025-03570-6

**Published:** 2025-05-21

**Authors:** Sameh Hefny, AbdelRahman El Gayar, Mostafa K. Ghobashy, Mohamed Elsayed Youssef, Mohamed AR AbdelFatah, Khaled Elshazly

**Affiliations:** Neurosurgery Department, Faculty of Medicine, Ain Shams University, Cairo, Egypt

**Keywords:** Transforaminal lumbar interbody fusion, Lumbar cage, Cage subsidence, Risk factor

## Abstract

Cage subsidence is a common complication after transforaminal lumbar interbody fusion (TLIF), leading to gradual loss of disc height and foraminal restenosis. The effect of cage size on early postoperative cage subsidence has barely been investigated. This study aimed to determine if there is an association between the size of the transforaminal lumbar cage and cage subsidence. This retrospective cohort study included patients who underwent single-level open TLIF at our tertiary hospital from 2018 to 2023. We collected demographics, comorbidities, preoperative data, and follow-up notes for one year. We defined cage subsidence as loss of disc height by more than 2 mm at the fusion level one year after TLIF. Our study included 81 cases with an average age of 45.12 years, including 37 males (45.67%). At the 1-year follow-up, cage subsidence occurred in 17 patients (20.98%). We divided the included cases into two groups: group (A) Cage non-subsidence group (64 cases) and group (B) Cage subsidence group (17 cases). The two groups had no significant difference in preoperative clinical and radiological variables. There was no significant difference between the two groups regarding the cage size. The fusion rate was statistically higher in the non-subsidence group. We didn’t find an association between the size of the lumbar cage and cage subsidence one year after TLIF, suggesting that cage size is not a risk factor for cage subsidence. Future studies should focus on integrating bone quality assessment and surgical technique refinement.

## Introduction

Degenerative lumbar spinal diseases are increasing and carry a wide range of symptoms and disabilities that compromise the quality of life [1]. Transforaminal lumbar interbody fusion (TLIF) is a common surgical procedure for spondylolisthesis, recurrent disc prolapses, lumbar canal stenosis, and post-decompression instability [2]. Placing a cage in the disc space promotes solid fusion, maintains proper disc height with indirect foraminal decompression, and restores the sagittal alignment [3].

Pedicle screw fixation combined with the transforaminal lumbar interbody cage results in high fusion rates in patients with lumbar degenerative diseases [4]. However, the placement of a transforaminal lumbar cage can result in several operative and postoperative complications. One of the common postoperative complications is cage subsidence towards the adjacent vertebral endplates. Progression of cage subsidence results in a gradual loss of disc height and foraminal restenosis and disturbs the sagittal alignment, which could impact clinical outcomes [5].

Cage subsidence may be related to many factors, like the surgical technique and the vertebral bone quality [6]. To our knowledge, the studies that evaluated the effect of cage size on early postoperative cage subsidence are deficient. This study aimed to determine if there is an association between the size of the transforaminal lumbar cage and cage subsidence.

## Materials and methods

In writing this retrospective cohort study, we followed the STROBE “Strengthening the Reporting of Observational Studies in Epidemiology” principles. We performed this research following the Code of Ethics of the World Medical Association (Declaration of Helsinki) for human studies. The research ethics committee of our faculty of medicine (FWA 000017585) approved our study protocol.

We reviewed the medical records at our tertiary hospital from January 1, 2018, to July 1, 2023, searching for patients with degenerative lumbar diseases in L3-4, L4-5, or L5-S1. We included the patients who underwent single-level open TLIF and followed up for at least 12 months in our outpatient clinic. Our spine surgeons obtained informed consent from the treated patients and agreed to use their medical data anonymously for research. We collected demographics, comorbidities, preoperative clinical status, radiological findings, intraoperative data, postoperative complications, and follow-up notes. The back pain and sciatica were assessed by visual analogue score (VAS), and the functional disability was evaluated by the Oswestry Disability Index (ODI).

**Fig. 1:**
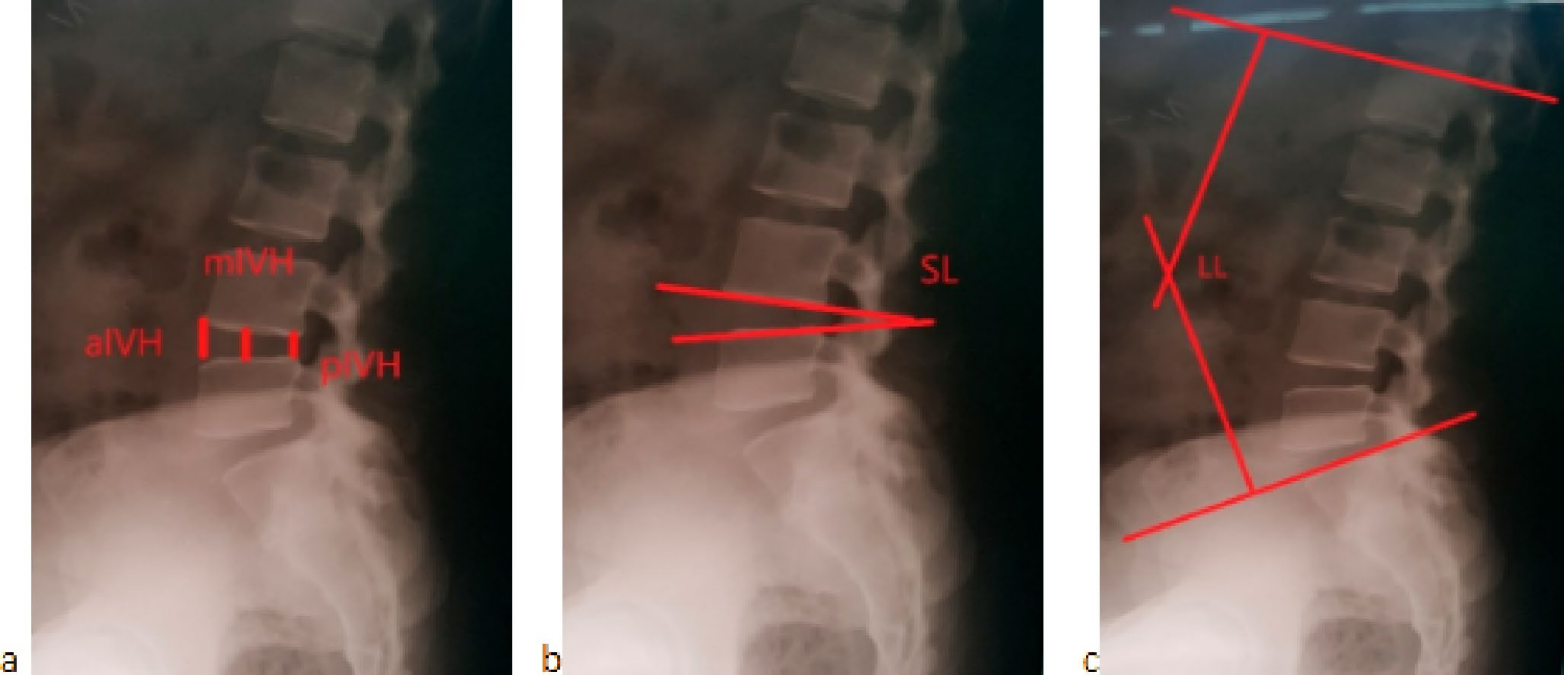
Plain X-rays on the lumbosacral spine lateral views showing **a**-measurement methods of the pathological segment’s anterior, posterior, and midpoint intervertebral height (aIVH, pIVH, and mIVH), **b**- measurement of segmental lordosis (SL) **c**- measurement of lumber lordosis (LL)

We measured the intervertebral disc height on a standing plain X-ray lumbosacral spine lateral view. We drew three lines extending vertically from the anterior and posterior edges and the midpoint of the superior endplate toward the inferior endplate. These lines define the anterior intervertebral height (aIVH), posterior intervertebral height (pIVH), and middle intervertebral height (mIVH), respectively, as seen in Fig. 1. We calculated the disc height using the following equation: $\:\frac{\mathrm{a}\mathrm{I}\mathrm{V}\mathrm{H}+\:\mathrm{p}\mathrm{I}\mathrm{V}\mathrm{H}+\:\mathrm{m}\mathrm{I}\mathrm{V}\mathrm{H}\:}{3}$

Segmental lordosis (**SL**) is the angle between the superior and inferior endplates of the pathological disc level. For evaluating the lumbar lordosis (**LL**), the Cobb angle is measured by drawing a line through the upper margin of the first lumbar vertebra and a second line through the upper endplate of the first sacral vertebra, as shown in Fig. 1. We measured the Hounsfield unit (HU) on the CT scan to determine the bone quality. Four values of Hounsfield units were measured across the index disc space (just above and below the end plates and in the middle of the adjacent vertebral bodies), and we recorded the mean value of the four parameters as the mean HU value.

The spine team of neurosurgeons in our university hospital carried out all the TLIF procedures and the bilateral pedicular screws fixation. A Capstone^®^ PEEK (polyetheretherketone) interbody cage (Medtronic Sofamor Danek) was inserted in all cases. All the cages were placed centrally in the disc space. The cages were filled with autologous bone grafts and demineralized bone matrix. Intraoperative fluoroscopy verified the proper alignment of the cages and the satisfactory disc heights. The placed pedicle screws were polyaxial and from a single brand. The operative segment’s upper and lower bony endplates were preserved.

We defined fusion as the presence of trabecular bridging bone connecting the adjacent vertebral bodies. We assessed fusion one year after TLIF via a thin-cut CT scan sagittal lumbar reconstruction. Three senior spine surgeons divided the fusion rates into good, accepted, and poor. We defined cage subsidence as loss of disc height by more than 2 mm at the fusion level one year after TLIF [7].

### Statistical analysis

We used SPSS (version 19.0; IBM Corp., Armonk, NY, USA) for data analyses. The values were expressed by mean ± standard deviation following normality analysis. We divided the included cases into two groups according to cage subsidence: the cage non-subsidence group and the cage subsidence group. We compared continuous data using the independent t-test. Categorical data were shown as numbers and frequencies and compared using the chi-square test. A p-value **<** 0.05 was determined as statistically significant.

## Results

Our study included 81 cases who underwent single-level TLIF with bilateral pedicular screw fixation. The average age of the included patients was 45.12 years (ranging from 29 to 68 years), including 37 males (45.67%) and 44 females (54.33%). The median follow-up was 17 months (13 to 26 months). At the 1-year follow-up, we found that cage subsidence developed in 17 patients (20.98%). Thus, we divided the included patients into two groups: group (A) cage non-subsidence group (**64 cases**) and group (B) cage subsidence group (**17 cases**).

Table 1 lists the demographics and comorbidities of the included patients. Table 2 shows their preoperative diagnoses. There was no significant difference between the two groups in age, gender, body mass index, smoking history, previous surgery at the index pathological disc level, comorbidities, and preoperative diagnosis.

**Table 1:** The demographics and comorbidities of the included patients

	Non-subsidence Group (*n* = 64)	Subsidence Group (*n* = 17)	*P*-value
Age (years)			
Mean ± SD	44.87 ± 7.63	45.88 ± 8.31	0.89
Range	29–62	43–68	
Gender			
Male	30 (46.9%)	7 (41.2%)	0.09
Female	34 (53.1%)	10 (58.8%)	
BMI (kg/m^2^) ± SD range	26 ± 3.47 (19–32)	26.67 ± 3.31 (19–31)	0.9
Cigarette Smoking	19 (29.68%)	5 (29.41%)	0.9
Previous surgery at the index level	18 (28.12%)	7 (41.17%)	0.29
Comorbidities			
Hypertension	4 (6.25%)	2 (11.7%)	0.43
Diabetes	10 (15.6%)	3 (17.6%)	0.19
Myocardial ischemia	3 (4.68%)	1 (5.88%)	0.48

BMI: average body mass inde × SD: standard deviation

**Table 2:** The preoperative diagnosis of the included patients

Preoperative diagnosis	Fused Levels	Non-subsidence group (*n* = 64)	Cage subsidence group (*n* = 17)	*P*-value
Recurrent disc prolapse	L3-4 L4-5 L5-S1	4 7 7	1 4 2	0.7
Degenerative spondylolisthesis	L3-4 L4-5 L5-S1	2 9 12	0 2 3	
Isthmic spondylolisthesis	L4-5 L5-S1	6 5	1 1	
Lumbar spinal canal stenosis	L3-4 L4-5	6 6	1 2	

Table 3 shows the pre- and postoperative clinical and radiological variables. The two groups had no significant difference in preoperative clinical and radiological variables. The postoperative improvement in back pain was significantly better in the non-subsidence group, while there was no significant difference regarding the improvement of postoperative sciatica. In addition, the postoperative functional score was statistically better in the non-subsidence group. The one-year postoperative mean Hounsfield units decreased in all the included patients, however, there was no significant difference between the two groups.

**Table 3:** The pre- and postoperative clinical and radiological variables

Variables	Preoperative			One year after the operation		
	Non-subsidence group (*n* = 64)	subsidence group (*n* = 17)	*P*-value^§^	Non-subsidence group (*n* = 64)	subsidence group (*n* = 17)	*P*-value^§^
	Mean ± SD (range)			Mean ± SD (range)		
Back pain VAS	6.87 ± 1.18 (4–9)	6.81 ± 1.15 (4–9)	0.78	2.31 ± 0.9 (1–6)	3.92 ± 0.62 (2–7)	0.01*
Sciatica VAS	7.87 ± 1.67 (4–10)	8 ± 1.45 (4–10)	0.82	1.03 ± 1.02 (0–3)	1.06 ± 1.12 (0–3)	0.89
ODI	67.62 ± 13.01	69.81 ± 13.71	0.74	17.78 ± 5.08	21.56 ± 7.64	0.003*
Disc height (mm)	9.03 ± 2.19 (4–12)	8.93 ± 0.7 (4–12)	0.91	11.37 ± 1.17 (9–13)	10.18 ± 0.95 (8–12)	0.007*
Mean HU	151.62 ± 41.1	149.15 ± 36.8	0.3	146.0 ± 18.4	126.1 ± 16.7	0.8

^*^ statistically significant ^§^ Paired samples T-test. VAS: visual analogue score

ODI: Oswestry Disability Index HU: Hounsfield unit

Table 4 illustrates the preoperative, immediate postoperative, and one-year postoperative segmental and lumbar lordosis. The two groups had no significant difference in the immediate postoperative segmental and lumbar lordosis. One year postoperatively, segmental and lumbar lordosis were significantly better in the cage non-subsidence group.

**Table 4:** The preoperative and postoperative segmental and lumbar lordosis

	Segmental lordosis		*P*-value^§^	Lumbar lordosis		*P*-value^§^
	Non-subsidence group (*n* = 64)	Subsidence group (*n* = 17)		Non-subsidence group (*n* = 64)	Subsidence group (*n* = 17)	
Preoperative	8.62° ± 1.37 (5°-11°)	8.75° ± 1.15 (7°-11°)	0.23	20.8° ± 1.73 (17°-25°)	20.62° ± 1.7 (18°-24°)	0.4
Immediate postoperative	16.93° ± 1.76 (14°-20°)	17.04° ± 1.82 (14°-21°)	0.67	39.1° ± 4.02 (32°-46°)	38.9° ± 3.97 (31°-47°)	0.5
One year postoperative	13.37° ± 1.45 (10°-16°)	11.25° ± 1.46 (9°-15°)	0.03*	34.3° ± 4.04 (28°-43°)	28.25° ± 1.38 (26°-35°)	0.01*

^*^ statistically significant ^§^ Paired samples T-test

Table 5 illustrates the operative findings, postoperative complications, and fusion rates. Both groups had no significant difference in pathological levels, mean operative time, and average blood loss. There was no significant difference between the two groups regarding the cage size. The width and depth of all the inserted TLIF cages were constant (14 × 30 mm).

**Table 5:** The operative findings, postoperative complications, and fusion rate

		Non-subsidence group (*n* = 64)	Subsidence group (*n* = 17)	*P*-value
**Levels**	L3-4	12 (18.8%)	2 (11.76%)	0.7
	L4-5	28 (43.75%)	9 (52.9%)	
	L5-S1	24 (37.5%)	6 (35.29%)	
**Mean Operative time**		147.2 ± 19.12 min	144 ± 18.75	0.97
**Blood loss (ml)**		302.8 ± 63.1	283.1 ± 61.3	0.94
**Cage size (mm)**		12.5 ± 1.2 (10–14)	12.3 ± 1.1 (10–14)	0.89
**Cage width (mm)**		14	14	
**Cage depth (mm)**		30	30	
**Fusion**				
Good		60 (93.75%)	13 (76.4%)	0.01*
Accepted		4 (6.25%)	2 (11.8%)	
Poor		0 (0%)	2 (11.8%)	
**Complications**				
Wound infection		1 (1.56%)	0	0.76
Motor weakness		1 (3.1%)	0	

^*^ statistically significant

The fusion rate was statistically higher in the non-subsidence group. None of the included patients required revision surgery within 1 year after TLIF.

There was no statistical difference between the two groups in terms of complications. In the non-subsidence group, one patient developed superficial wound infections, which responded to conservative treatment. Another patient suffered from immediate postoperative deterioration in motor power of unilateral foot dorsiflexion from grade 4 to grade 3, mostly from root manipulation. Immediate CT LSS was performed, and no intervention was required. The weakness improved to grade 5 one month later.

## Discussion

We aimed to determine if there is an association between the size of the transforaminal lumbar cage and cage subsidence. At the 1-year follow-up, 17 patients (20.98%) developed cage subsidence. We divided the included patients into two groups: group (A) cage non-subsidence (64 cases) and group (B) cage subsidence (17 cases). Zhang et al. reported 34.1% cage subsidence at one-year follow-up after TLIF. However, they included only patients over 55 years, and 48% were above 70 [1].

Our groups showed no significant difference in age, gender, previous surgery at the index level, comorbidities, or preoperative clinical and radiological variables. Similarly, Zhang et al. found no significant association between age and cage subsidence 1 year after TLIF [1]. Additionally, both groups had no significant difference in operative time and average blood loss.

Theoretically, the risk of cage subsidence increases as the cage height increases. However, we didn’t find a significant association between the size (height) of the lumbar cage and cage subsidence one year after TLIF. Contrary to our findings, Singhatanadgige et al. emphasized that a cage height ≥ 12 mm was a risk factor for cage subsidence based on their study of patients who underwent minimally invasive TLIF [8]. We recommend ensuring proper disc height for indirect foraminal decompression and restoring sagittal alignment, irrespective of the size of the lumbar cage.

Endplate integrity is a key determinant in preventing cage subsidence [9]. While larger cage footprints (depth and width) provide better load distribution and are expected to reduce stress per unit area, early subsidence can occur if the endplate is compromised due to low bone mineral density. Similarly, smaller cage footprints, while minimizing surgical disruption, may increase focal stress on a limited surface area, exacerbating subsidence, especially in osteoporotic patients [10].

Excessive violation of the subchondral bone during endplate preparation weakens structural support, making even a well-sized cage susceptible to subsidence into the vertebral body. The technique used in disc space preparation could significantly influence the mechanical stability of the cage within the intervertebral space [11]. Moreover, in an attempt to restore disc height, surgeons may inadvertently create excessive tension on the endplates, particularly when using larger cage heights, leading to stress fractures upon cage insertion. Choosing an appropriately sized cage that balances load distribution without excessive endplate pressure is critical.

A conservative approach that ensures minimal endplate violation and maintains the integrity of the subchondral bone can reduce the risk of cage subsidence. Understanding the interaction between cage size, endplate integrity, and disc preparation can inform surgical decision-making and reduce the risk of subsidence. Thus, during preoperative planning, CT-based assessment of bone mineral density and dual-energy x-ray absorptiometry (DEXA) can help select an appropriate cage size and anticipate the need for additional reinforcement techniques.

The improvement in postoperative back pain and functional status was significantly better in the non-subsidence group. However, there was no significant difference between the two groups in terms of postoperative sciatic pain. This lack of difference may be related to the adequate decompression of the traversing and exiting nerve roots for placing the transforaminal lumbar cage.

We found that one-year postoperative segmental lordosis, lumbar lordosis, and disc height were statistically better in the cage non-subsidence group. Zhou et al. reported that cage subsidence results in a gradual loss in SL and LL [12]. Similarly, Kong et al. emphasized that anterior column reconstruction is prone to failure with cage subsidence [13].

Xie et al. stated that the preoperative HU value could be considered an effective predictor of lumbar cage subsidence. However, in our study, there was no significant difference in the pre- and postoperative mean HU between the two groups. We found a statistically higher fusion rate in the non-subsidence group. However, Zhou et al. found no relation between fusion rate and cage subsidence [12]. There was no significant difference between the two groups in terms of complications.

The main strengths of this study are the precise definitions and standardized outcome measures that ensure reliable and reproducible results, the single cage model with fixed width and depth, eliminating variability in cage footprint as a confounding factor for cage subsidence, and the inclusion of a relatively wide age range of patients. We included only the single-level TLIF procedures performed by the same spine team of neurosurgeons to limit the operative variabilities between different spine teams. However, while limiting these potential variables, the sample size became relatively small, which might affect the power of our study. Also, the limited variables could affect the generalizability of the results.

In our study, the narrow cage height range (10–14 mm) could mask an association with cage subsidence due to the limited variability in mechanical loading and endplate contact characteristics. The short follow-up period captured early outcomes but is insufficient for assessing delayed subsidence, long-term fusion stability, or revision rates. Also, we did not evaluate osteoporosis, as dual-energy x-ray absorptiometry was not routine.

Large-sample-size studies with longer follow-ups are required to determine the risk factors of lumbar cage subsidence and its impact on clinical outcomes. Future studies should focus on integrating bone quality assessment and surgical technique refinement to enhance interbody fusion success rates.

## Conclusion

We didn’t find an association between the size of the lumbar cage and cage subsidence one year after TLIF, suggesting that cage size is not a risk factor for early cage subsidence. Large-sample-size studies with longer follow-ups are required to determine the risk factors of cage subsidence and its impact on clinical outcomes. Future studies should focus on integrating bone quality assessment and surgical technique refinement to enhance interbody fusion success rates.

## Data Availability

No datasets were generated or analysed during the current study.
